# Real-Time Acoustic Detection of Critical Incidents in Smart Cities Using Artificial Intelligence and Edge Networks

**DOI:** 10.3390/s25082597

**Published:** 2025-04-20

**Authors:** Ioannis Saradopoulos, Ilyas Potamitis, Stavros Ntalampiras, Iraklis Rigakis, Charalampos Manifavas, Antonios Konstantaras

**Affiliations:** 1 Department of Electronics, Hellenic Mediterranean University, 73133 Chania, Crete, Greece; ddk86@edu.hmu.gr (I.S.); rigakis@hmu.gr (I.R.); akonstantaras@hmu.gr (A.K.); 2 Department of Music Technology & Acoustics, Hellenic Mediterranean University, 74133 Rethymno, Crete, Greece; 3 Department of Computer Science, University of Milan, 20133 Milan, Italy; stavros.ntalampiras@unimi.it; 4 Institute of Computer Science, Foundation for Research and Technology—Hellas, 70013 Heraklion, Crete, Greece; harryman@ics.forth.gr

**Keywords:** acoustic surveillance, IoT, transformers, edge computing, AudioSet

## Abstract

We present a system that integrates diverse technologies to achieve real-time, distributed audio surveillance. The system employs a network of microphones mounted on ESP32 platforms, which transmit compressed audio chunks via an MQTT protocol to Raspberry Pi5 devices for acoustic classification. These devices host an audio transformer model trained on the AudioSet dataset, enabling the real-time classification and timestamping of audio events with high accuracy. The output of the transformer is kept in a database of events and is subsequently converted into JSON format. The latter is further parsed into a graph structure that encapsulates the annotated soundscape, providing a rich and dynamic representation of audio environments. These graphs are subsequently traversed and analyzed using dedicated Python code and large language models (LLMs), enabling the system to answer complex queries about the nature, relationships, and context of detected audio events. We introduce a novel graph parsing method that achieves low false-alarm rates. In the task of analyzing the audio from a 1 h and 40 min long movie featuring hazardous driving practices, our approach achieved an accuracy of 0.882, precision of 0.8, recall of 1.0, and an F1 score of 0.89. By combining the robustness of distributed sensing and the precision of transformer-based audio classification, our approach that treats audio as text paves the way for advanced applications in acoustic surveillance, environmental monitoring, and beyond.

## 1. Introduction

In recent years, the demand for advanced surveillance systems has surged, driven by the need for enhanced security and situational awareness in urban environments. Traditional surveillance methods, which are primarily reliant on visual data, often encounter limitations in low-light conditions or obstructed views. To address these challenges, integrating audio surveillance has emerged as a complementary approach, offering the ability to detect and classify sounds that may indicate security events. The audio modality is more affordable than a camera and requires less power and bandwidth when transmitting data. Additionally, microphones have greater scalability potential to be deployed across large areas, are relatively inexpensive compared to cameras, and are less invasive than video surveillance.

The topic of audio surveillance has a long history, where different classifiers such as Gaussian mixture models and hidden Markov models [1], and support vector machines have been applied to classify audio recordings (see [2,3,4] for previous approaches of ours and the references therein). At that time, shallow classifiers were taken mostly from the speech recognition paradigm and were applied to private and limited to a narrow domain audio dataset that would not allow the advancement of the field. For a broader review of acoustic methods in audio surveillance applications, see [5,6,7,8,9]. From our perspective, two key factors now enable us to approach acoustic surveillance from a more pragmatic and applied standpoint: (a) the availability and openness of a large audio database equivalent to ImageNet for images—namely, AudioSet [10,11], which contains more than 2 million labeled recordings—and (b) the advent of transformer technology, which has surpassed deep learning architectures [12] in recognition performance, achieving scores that make the development of commercial audio surveillance products feasible. With AudioSet and transformers, the robustness and reliability barrier [13,14] required for real-world applications has now been overcome.

The development of real-time, distributed audio surveillance systems necessitates the convergence of multiple technologies, including edge computing platforms, efficient data transmission protocols, encryption, audio processing and artificial intelligence (AI). Edge devices, such as ESP32 platforms, are increasingly utilized for their low power consumption and wireless communication capabilities, making them suitable for deploying distributed sensor networks. These devices can capture audio data from multiple distributed locations and transmit it to more powerful processing units for audio classification.

Central to the processing of audio data is the application of machine learning models capable of accurately classifying and interpreting sounds. Transformers, a class of models originally designed for natural language processing, have been adapted for vision. Subsequently, through the spectrogram, the audio is turned into a picture on which vision-based transformers are adapted for audio tasks, demonstrating significant improvements in performance. Notably, the Audio Spectrogram Transformer (AST) has been trained on large-scale datasets like AudioSet, enabling it to effectively classify 632 audio classes [15].

The contribution of this work is twofold: (A) We provide details of a platform that integrates diverse technologies from distinct research domains into a practical system, enabling experimentation and evaluation of new audio-recognition architectures. We present the implementation of a real-time, distributed audio surveillance system that incorporates ESP32-based microphones, audio compression, MQTT communication, transformer-based audio classification embedded in Raspberry Pi processing units, encryption protocols, and large language model (LLM)-driven graph analysis. (B) By structuring classified audio events into graph representations, we propose a novel approach for querying hazardous incidents hierarchically within rolling timeframes, reducing both missed critical incidents and false alarms. We compare our approach to LLaMA-3.1-8B, which has access to the same AST labels and is prompted on a per-chunk basis to determine whether a hazardous incident occurs (e.g., a car crash). The system’s effectiveness is evaluated through a series of real-world experiments, demonstrating its potential for deployment in various surveillance scenarios. To assess accuracy, precision, and recall, we analyze the audio from a 1 h and 40 min long movie, where our approach achieves an accuracy of 0.882, precision of 0.8, recall of 1.0, and an F1 score of 0.89.

Our goal is to make audio surveillance widespread to enhance safety and operational efficiency across various sectors such as borders, airports, government buildings, schools, sports and entertainment venues, metro lines, houses of worship, streets, and city networks [16]. However, the essence of surveillance is to discover a targeted signal in a cluttered world and the same technology can be used to diverse applications of monitoring after the transformer model is adapted to the audio or vibrational data of the specific application context. For example, biodiversity and conservation applications [17,18] for both terrestrial [19] and maritime [20] animals fall within the same paradigm. In a broader sense, machine condition monitoring [21,22] involves the use of vibratory queues to detect and prevent unintentional threats or hazards that could harm property and people in their working environment. Last and literally not least, the detection of respiratory diseases [23,24] are also special cases of acoustic surveillance.

## 2. Materials and Methods

A platform that integrates multiple audio sensing nodes must address several technical challenges. The nodes are required to continuously transmit audio clips to the base system, necessitating carefully optimized C programming to capture, compress, and encrypt audio at the node level. Communication with the nodes must be bidirectional to enable remote reconfiguration. The gateway system, which collects and classifies audio events, requires multi-threaded programming. One thread should unobtrusively collect and preprocess a queue of recordings, while another thread classifies the clips. This process must be as fast as possible to prevent clips from accumulating in the queue.

Additional security measures are implemented on the gateway as it deals with data that raise ethical issues. The gateway is also responsible for visualizing historical data, enabling queries, and providing telemetry for the system. Lastly, the gateway must parse the database that stores individual graphs for all nodes to identify incidents at both the local and collective levels of the sensing nodes.

### 2.1. Hardware Components

**Figure 1:**
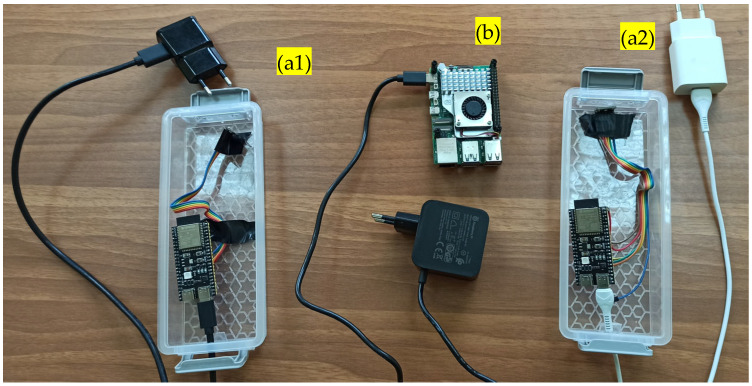
Hardware components used in the audio surveillance application. At the center, a Raspberry Pi 5 receives audio snippets from two ESP32-S3 nodes (**left** and **right**) denoted by (**a1**) and (**a2**). It is denoted by (**b**). Each ESP32-S3 is equipped with an SPH0645LM4H MEMS microphone and transmits audio snippets via Wi-Fi. They are both denoted by (**a**) because they are identical.

We implemented a distributed architecture that supports multiple deployment models, including cloud-based, on-premises, and edge computing models [25,26]. A network of ESP32-S3-DevKitC-1-N8R8 nodes from Espressif Systems, Shanghai, China, bearing a SPH0645LM4H microphone from Knowles, Itasca, IL, USA, transmit compressed audio snippets through Wi-Fi to a base station for classification. We tried using different platforms as base stations, including Raspberry Pi 4/5 from Raspberry Pi Holdings plc, Cambridge, England and an i5 laptop model HP 250g8: Hewlett Packard, Palo Alto, CA, USA. See Figure 1 for the details of the edge devices used. All signal processing and pattern recognition are performed on the edge devices, with only the recognized and timestamped incidents uploaded to a server.

The SPH0645LM4H-1 is a miniature, low-power, bottom port microphone with an I2S digital output. The solution consists of a proven high-performance acoustic sensor, a serial Analog-to-Digital convertor, and an interface to condition the signal into an industry-standard 24-bit I2S format. The I2S interface simplifies the integration in the system and allows it to directly interconnect with microcontrollers. Eliminating the need for an external audio codec, the SPH0645LM4H-1 is suitable for portable applications where size and power consumption are constraints. It has a flat Frequency Response between 0.1 and 8 kHz which is the frequency range we are interested in, since the AST expects to receive 16 kHz signals.

ESP32-S3 (see Figure 1a) is a general-purpose Wi-Fi and Bluetooth LE MCU energy-efficient microcontroller that integrates complete Wi-Fi and Bluetooth LE functions. It features a dual-core Xtensa LX7 processor, built-in Wi-Fi and Bluetooth 5 (LE), and enhanced support for vector instructions to accelerate machine learning tasks. The ESP32-S3 offers plenty of GPIOs and support for SPI, I2C, UART, and I2S interfaces, making it suitable for IoT, smart home, and audio processing applications. With its integrated AI capabilities, expanded PSRAM options, and improved security features, the ESP32-S3 was our choice for our embedded microphone system and MQTT handling of recordings.

The Raspberry Pi 5 (see Figure 1b) is a single-board computer with a 2.4 GHz quad-core Arm Cortex-A76 processor, improved GPU, and support for PCIe expansion. It is suitable for processing audio signals from ESP32-S3 nodes, enabling real-time data streaming, feature extraction, and classification. When integrated with ESP32-S3 nodes for audio capture, it facilitates MQTT-based transmission, preprocessing, and inference using machine learning models. The device includes high-speed USB and Gigabit Ethernet, supporting low-latency audio processing and data management in distributed systems.

### 2.2. Software Components

#### 2.2.1. Software at the Nodes

The ESP32-S3 Audio Recording System (see Figure 1a) is based on the SDK espressif esp-idf 5.3 and captures audio from a microphone using the I2S interface. It records continuously non-overlapping windows of 1024 samples at 16 kHz, processes the audio to check volume levels (RMS), encodes it into MP3 format, and transmits the data via MQTT to a remote gateway. Its architecture leverages the dual-core functionality of the ESP32-S3, with Core 0 handling audio capture and processing, and Core 1 managing encoding and data transmission. A ring buffer is used for inter-task communication. The system is configurable through a non-volatile storage (NVS) settings file, supports secure MQTT connections with TLS, allows dynamic configuration via MQTT messages, and employs pseudostatic read-only memory (PSRAM) for efficient memory management. It connects to the network via Wi-Fi but can be configured to cooperate with GPRS and supports remote configuration.

We embedded the Lame MP3 encoder (libMP3lame_for_esp32) in the ESP32-S3 nodes and a ffmpeg decoder in the Raspberry Pi platforms. The MQTT included in the espressif esp-idf 5.3 takes on the flow of the recordings to the base station and the error handling of packets. Regarding communication protocols, the primary reason to choose MQTT is its ability to operate efficiently in environments where bandwidth is limited, network reliability is inconsistent, or devices have low processing power to ensure timely delivery of audio data for classification. The MQTT directs the audio chunks in a pool where they are lined and processed in turn by the second thread running the AST classifier.

Potential communication issues between audio recording ports and the audio stream management server have been mitigated and we took action to ensure data integrity and minimal latency. The procedure is described below.

The initial approach was to have all devices connected to the same Wi-Fi network to ensure low latency. The system optimizes low-latency audio streaming from ESP32-S3 devices using MQTT for parallel transmission management. A multi-threaded MQTT client processes incoming data streams efficiently, with Quality of Service (QoS) adjustments to balance reliability and latency. To minimize packet loss and jitter, buffering mechanisms and error detection techniques were implemented, while reconnection protocols ensure network resilience by allowing rapid recovery from disconnections. Latency reduction was achieved by optimizing packet size and transmission intervals, using I2S DMA buffering for efficient audio handling, and dynamically adjusting sampling rates based on network conditions. A UDP-based fallback mechanism is employed in cases where MQTT introduces excessive delays. The network configuration prioritizes 2.4 GHz Wi-Fi for improved range and stability, and local MQTT brokers prevent delays associated with WAN hops. Server-side optimizations include asynchronous, multi-threaded audio stream processing to prevent bottlenecks, buffering thresholds to maintain real-time performance, and NTP-based synchronization to ensure temporal consistency across multiple ESP32 devices. These combined strategies enhance the system’s efficiency, ensuring reliable, low-latency operation in real-world deployments.

#### 2.2.2. Software at the Gateway

**Figure 2:**
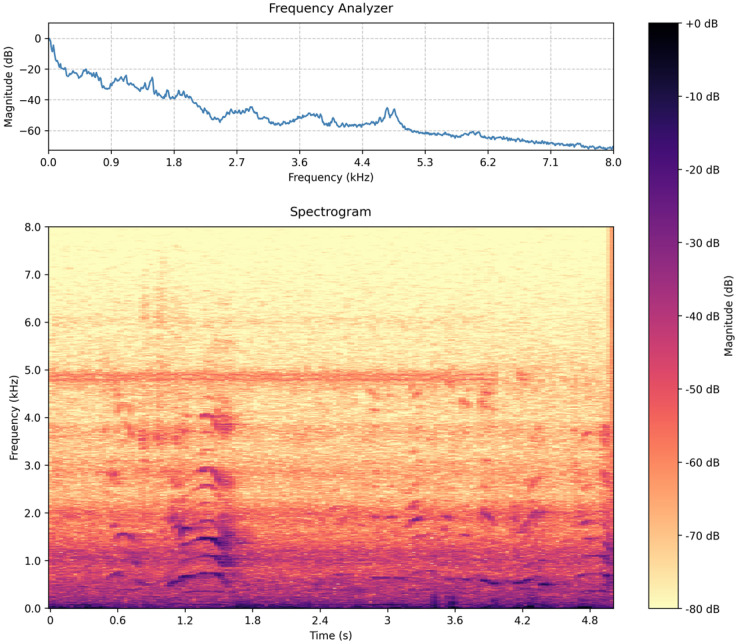
(**Top**): Spectral density estimation (Welch’s method) is a smoothed estimation of how the power of the signal is distributed over frequencies. (**Bottom**): Once the microphone is triggered, a 5 s snippet is recorded that is subsequently turned into a picture through the short-time Fourier transform (spectrogram). The spectrogram is a time-frequency representation (i.e., a 2D image) that serves as an input to the transformer model.

The gateway receives MP3 clips from all nodes and preprocesses them to become images for the transformer. A spectrogram is a visual representation of the frequency content of an audio signal as it varies over time. It is a two-dimensional plot where the *x*-axis represents time, the *y*-axis represents frequency, and the color intensity indicates the magnitude or energy of each frequency component at a given moment. Spectrograms are generated by applying a short-time Fourier transform (STFT) to divide the audio signal into overlapping segments and analyze their frequency content. This time-frequency representation takes the one-dimensional audio signal and creates a picture. It is widely used in speech processing, music, and environmental sound analysis for identifying patterns, understanding spectral dynamics, and recognizing features like harmonics, formants, and transient events (see Figure 2 for a snippet of spoken language).

Transformers are a neural network architecture designed for processing sequential data. They use linear algebra to make a self-attention mechanism to compute the relationships between all elements in an input sequence simultaneously, avoiding the sequential dependencies inherent in recurrent neural networks. The architecture consists of stacked encoder and decoder layers, with each layer comprising multi-head self-attention mechanisms and feedforward neural networks. Positional encodings are added to the input embeddings to provide the model with information about the order of the sequence. Transformers are computationally efficient for parallel processing due to their non-sequential design and are highly scalable to large datasets [26]. The Audio Spectrogram Transformer (AST) adapted the Vision Transformer (ViT) architecture [27] for audio processing by converting audio signals into spectrogram images. This model achieves state-of-the-art performance in audio classification tasks. AST [15] is designed to directly map audio spectrograms to their corresponding labels, achieving a mean average precision (mAP) of 0.485 on AudioSet, 95.6% accuracy on the ESC-50 dataset, and 98.1% accuracy on Speech Commands V2 [28].

We embedded the HuggingFace implementation in [29]. A Python 3.11.2 wrapper on the AST handles the communication between the compressed audio and the transformer itself. The AST is trained on the AudioSet database and requires the Raspberry Pi 5 to have an active cooling system for operation.

#### 2.2.3. The Training Database

AudioSet is a large-scale dataset for audio event recognition developed by Google and is extracted from Youtube videos. Though YouTube’s compression uses advanced audio codec (AAC/OPUS) and not MP3 compression, we observed a distinct improvement when we used compressed audio compared to uncompressed audio files. It contains over 2 million 10 s audio clips sourced from YouTube, annotated with a hierarchical ontology of 632 audio event classes. The dataset is designed for multi-label classification, where each clip can be associated with multiple event labels. AudioSet is widely used for training and benchmarking models in acoustic scene analysis, environmental sound classification, and other audio-related tasks [10,11,15,26].

### 2.3. Frontend Functionality

**Figure 3:**
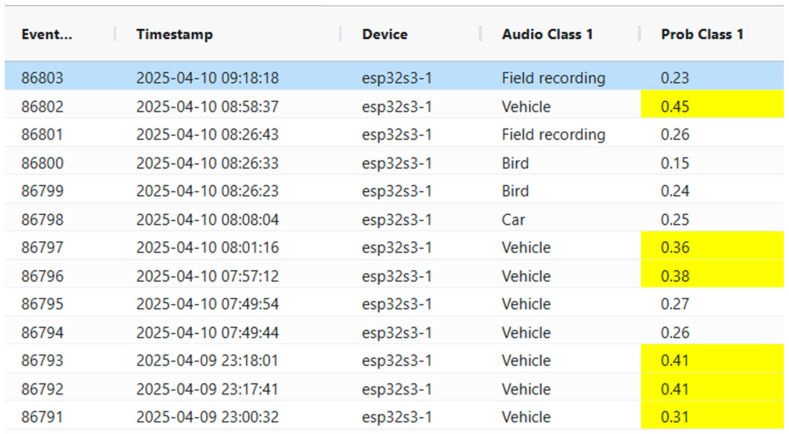
Audio-surveillance dashboard: events are sorted by time, devices are listed, and each audio event is automatically labeled by the transformer model. Color coding reflects the model’s confidence (probability of the label) in each classification.

**Figure 4:**
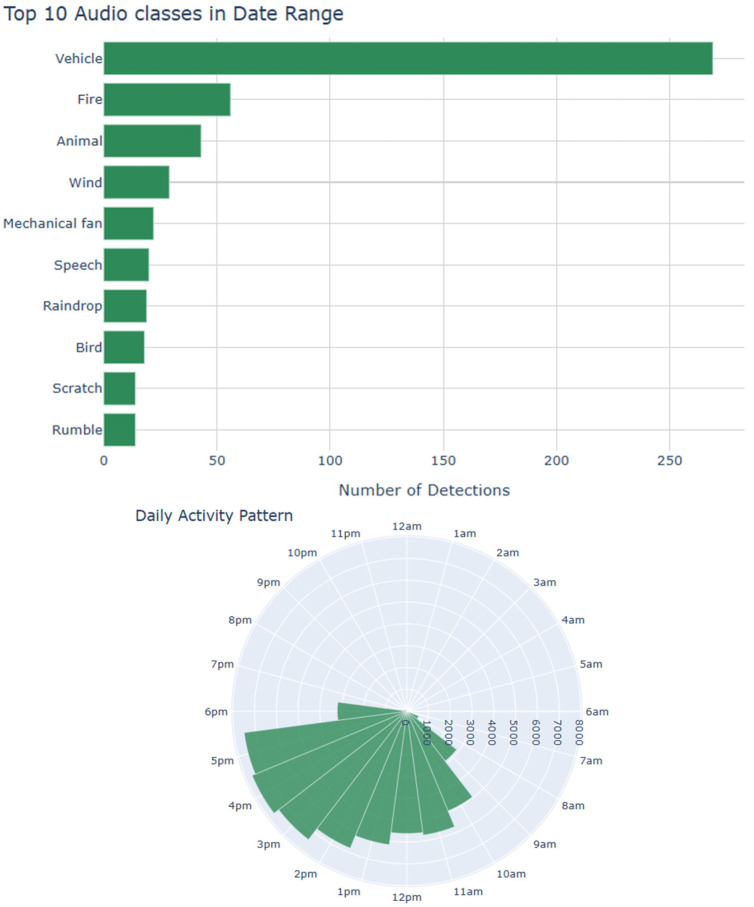
Data analytics. The database holds the labels produced by the AST and the timestamps of the audio events. This allows the creation of informative visualizations based on historical data. On the (**top**), we see the classes and at what rate they appear in a specific timespan, and on the (**bottom**), we can see a circular histogram of hourly activity. Classes of interest can be filtered and tracked down over time (**bottom** graph).

**Figure 5:**
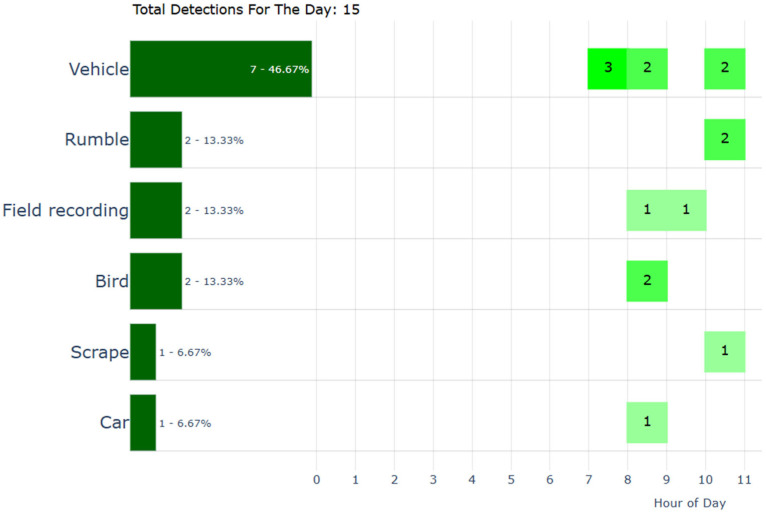
Hourly distribution of audio activity, showing the occurrence and frequency of different audio classes throughout a specific day. Only one part is displayed for clarity.

The user interface includes a user-friendly dashboard displaying real-time alerts, real-time event logs, and system status via telemetry. The frontend of the audio surveillance application is made in Dash v3.0.2 and JavaScript ECMAScript 2020. The Raspberry pi5 has embedded the AST and continuously monitors audio feeds from all ESP32 nodes to detect events as they happen. All detected events are logged with precise timestamps for accurate incident tracking, and the corresponding audio clips are included for verification and further analysis. Timestamps and classification results are maintained in a searchable SQL database of past events with filters for date, time, and event type (see Figure 3). The frontend triggers immediate alerts (e.g., push notifications, emails, SMS) when specific audio events are detected, such as alarms, gunshots, broken glass, footsteps, or screams. The users are allowed to set probability thresholds for alerts to minimize false positives. We use visual cues (i.e., color-coding) to represent the confidence level of detections so that events of interest are immediately discoverable by a human operator. The user can configure and filter which audio classes from the AudioSet are monitored based on the application scenario. Figure 4 presents statistics on class distributions within user-defined time spans and overall audio activity throughout the day. These visualizations enable users to select specific audio classes (e.g., bird vocalizations) and analyze historical data for targeted sound categories. Figure 5 illustrates hourly audio activity for a selected day, providing insight into daily events, which is particularly useful in contexts such as elderly care or a scenario involving industrial facility surveillance. All recorded events can be played back to manually verify class attribution. The database is exported in JSON format, and the frontend interface supports remote configuration of all reporting nodes, including adjustments for clip duration, compression rate, and the RMS threshold for audio activity detection.

### 2.4. Graph Parsing

Audio surveillance is a computer audition task also known as acoustic scene classification. It is a multi-class, clip-level classification problem and the task can have more than one label. The main challenges of audio surveillance in continuous operation are twofold: (a) incidents related to hazardous situations, whether unlawful actions or accidents, are rare compared to typical audio events encountered in everyday activities; and (b) false alarms are likely, as audio events under operational conditions occur at an average rate of one every 15 s. If not addressed, these false alerts can overwhelm the user with frequent notifications over time.

In our system, the recognized audio events can be exported in the form of a JSON file. The JSON file contains a list of events (see Table 1). Each event has a unique event ID, a timestamp (e.g., “2024-11-23 02:30:26”) and a set of audio events, each with a class (type of sound) and a probability (how likely that sound is to be present according to the AST). Using graphs to model events provides a structured way to understand temporal and spatial relationships, making it easier to track sequences or patterns of suspicious behavior.

The AST is quite accurate in its classifications, but a targeted incident cannot be based on a single label. For example, not every car sound or tire skidding results in a car crash. Honking may appear as an effort to warn other vehicles but is not a prerequisite in an accident. However, the impact sounds of vehicles colliding are a mandatory audio class. The aftermath of the collision can have many audio manifestations that also contribute to the total confidence score of the incident. Instead of just looking at isolated classes, we base our decision on clusters of audio events with key labels that must appear in a hierarchy and an accumulated score that surpasses a threshold (i.e., a two-stage screening of events). In the case of a car accident, we would need to identify the sequence of events leading to the collision. Given the JSON form of the database of AST-tagged audio events, we attempt to detect potential “incidents” from a timeline of recorded sound events. The structure is a particular pattern: first, there is a “primary” sound (like a car or squeal) that might suggest a vehicle’s presence, and then, within a short timeframe after that sound, a “secondary” sound (like breaking glass or a crash) that might indicate an accident or collision.

If such a pattern is found within a close timeframe (e.g., 5 s), the program flags it as a “potential incident.” For example, in a car accident, at least one of the primary sounds (‘car’, ‘truck’, ‘vehicle’, ‘bus’, ‘skidding’, ‘squeal’) and one of the secondary sounds (‘breaking’, ‘shatter’, ‘smash’, ‘crash’, ‘bang’, ‘thump’, ‘thud’, ‘slam’, ‘glass’) must coexist to register an incident. The primary class indicates vehicular presence and the secondary connotates a possible collision or accident. For a burglary incident, the primary class is (‘breaking’, ‘smash, crash’, ‘shatter’, ‘bang’, ‘glass’) and the secondary (‘walk, footsteps’, ‘creak’, ‘buzzer’, ‘siren’, ‘alarm’, ‘whispering’, ‘slam’). The custom dictionary of predefined sound categories is constructed manually from the ontology of the AudioSet and the sounds are grouped according to the context of a particular application (e.g., burglary, car crash, gunfire).

We analyzed several true car crashes found online, and all of them occurred within 5 s. Therefore, a rolling window with a configurable duration is necessary. For the burglary scenario, the time window is set to 1 min, and for gunfire, it is set to 5 s. Once these strict time constraints are defined, a window may contain from zero to a few relevant cases.

We retain the top three recognized audio classes along with their associated probabilities, as audio recognition models often exhibit some uncertainty, particularly in noisy environments or with overlapping sounds. Retaining the top three classes allows us to capture the model’s uncertainty and provide a more comprehensive view of possible events. If the top class’s probability is only slightly higher than the second, considering both can enhance the application’s reliability, especially in cases involving overlapping audio events. Occasionally, the targeted event may not appear with the highest probability due to temporal and/or frequency overlapping with another audio class.

All probabilities for the primary and secondary targeted audio top three classes within a short timeframe are accumulated to calculate a score for the primary and secondary labels separately. This score is then thresholded before the algorithm identifies an event as an “incident”.

The previous discussion can be summarized in a more detailed description of the algorithm that follows:

1. Define primary and secondary sound categories from the available AudioSet ontology.
2. Define the probability thresholds for primary and secondary labels.
3. Define the duration of time windows for identifying high-level events.
4. Read and parse the JSON file into a graph containing a list of labels of audio events. Events are sorted by their Device_ID and, subsequently, by their timestamps to enable time-sequential processing. Each event includes a timestamp, unique event ID, and associated audio events with class names and probabilities.
5. A rolling window of events is maintained for analysis. For each event, the audio events are filtered based on a probability threshold, and their classes are compared with the primary and secondary sound categories.
6. The primary and secondary scores are calculated by summing the probabilities of matching audio events within the time window. Older events outside the time window are removed from consideration.
7. If the total primary and secondary scores within the time window exceed predefined thresholds, a high-level event is identified. The events contributing to the high-level event are recorded, and their IDs are marked to avoid reprocessing. Subsequently, a classification object is created, including time window of detection, IDs of related events, classes of indicative audio events, and total primary and secondary scores. The classifications of detected high-level events are output to the console. For each high-level event, details such as the time window, related events, indicative classes, and scores are displayed.

To compare our incident-tagging method, we employed a large language model (LLM) to perform the same task—inferring incidents based on the same AST labels for a rolling window of audio events. Specifically, we used LLaMA-3.1-8B-Instruct by Meta [30]. For local execution, we utilized Llama.cpp, a lightweight and efficient implementation designed to run LLaMA-architecture models on both CPU and GPU without requiring specialized hardware. Written in C++, Llama.cpp supports multiple operating systems, including Windows, Linux, macOS, and Android, enabling efficient local execution of large language models with minimal resource consumption. We compiled Llama.cpp to support GPU acceleration, CUDA, and server capabilities [31]. Among the configurations we tested, the most efficient in terms of both response quality and execution time—given our hardware setup—is Llama-3.1-8B-Instruct with q8 quantization.

For interaction with the LLM and prompt engineering within our application, we employed DeepSpeed Prompting (DSPy) [32]. DSPy is a framework for prompt programming and language model fine-tuning, facilitating automated prompt selection, parameter tuning, and response optimization for large language models (LLMs).

In our latest crash test results, we utilized a sliding window of three chunks with a step size of one, based on the AST output. Each prompt within a window consists of a question, a context, and data from the three consecutive chunks. The prompting formulation can be found in the Appendix A.

### 2.5. Security Considerations

Audio recordings and subsequent classifications could be tampered with. Securing the data pipeline to prevent sensitive-data leakage and tampering or injection of fake audio is crucial. Attackers could intentionally manipulate the system by introducing misleading sounds or noises designed to confuse the classifiers or overwhelm the system.

To secure the communication chain using MQTT, we have followed several practical measures to ensure data confidentiality and system stability. First, we encrypted all communications between devices using MQTT over TLS (MQTTs). This prevents unauthorized interception of data during transit. On the ESP32 devices, the ESP-MQTT library, which supports TLS via mbedTLS, has been used, while for the Raspberry Pi, the Paho MQTT library has been configured for secure communication. We used the Mosquitto MQTT broker that supports TLS encryption. The broker has been configured to listen exclusively through secure ports, such as 8883, and to disable plain-text communication on ports like 1883.

Mutual TLS (mTLS) authentication provides an additional layer of security by ensuring that only trusted devices can communicate within the network. This involves generating unique X.509 certificates for each ESP32 device and the Raspberry Pi and configuring the MQTT broker to enforce certificate-based mutual authentication. Lightweight encryption, such as ECDHE-ECDSA-AES128-GCM-SHA256, has been applied; this is suitable for resource-constrained devices like the ESP32.

To protect the system’s stability against potential attacks, we implemented measures such as rate limiting and throttling on the MQTT broker to prevent abuse. This involves limiting the rate of incoming connections and the number of messages a client can send within a certain period. Additionally, payloads have been validated for size and structure both on the ESP32 devices and the Raspberry Pi to prevent attacks using malformed data. The MQTT broker has been further configured to limit the size of messages and enforce an Access Control List (ACL) to restrict the topics that each ESP32 can publish or subscribe to.

Replay attacks can be mitigated by including timestamps or nonces in the messages. On the Raspberry Pi, one can validate these timestamps and reject duplicate or outdated messages. Authentication and authorization can also be enhanced by using username–password pairs for each device and configuring the broker to enforce these credentials. To reduce the risk of denial-of-service (DoS) attacks, we have implemented measures such as limiting connection attempts per client IP and deploying a firewall as an intrusion prevention system on the Raspberry Pi.

In addition to securing communications, we deemed it essential to safeguard the firmware and credentials of our devices. The ESP32 supports secure boot, ensuring that only authenticated firmware is executed. Firmware updates can be delivered over HTTPS using secure over-the-air (OTA) mechanisms, while credentials can be stored securely in ESP32’s Non-Volatile Storage (NVS).

Raspberry Pi, which stores audio snippets and labels in a database, has also measures in place to secure its data. We encrypted the database using the sqlcipher3 0.5.4 library to ensure that data at rest remain protected. Access to the database was restricted to authorized applications, and authentication mechanisms like API keys were used.

Finally, monitoring and intrusion detection play an important role in maintaining system stability. Detailed logging of MQTT broker activity, such as connection attempts and message rates, helps detect potential issues. A lightweight intrusion detection system, Fail2Ban, has been deployed on Raspberry Pi5 to block suspicious activity. These combined measures create a robust framework for securing the MQTT communication chain while safeguarding against attacks that might compromise system stability.

### 2.6. Network of Nodes and Distributed Incidents

The prime role of a dispersed network of sensors is to provide the scale and the diversity needed for reliable discrimination of incidents. Sometimes, the targeted incident is a single event localized in space, e.g., a street gunshot or an explosion in an industrial facility. That event can be picked up by many sensors at slightly different times due to the propagation delay. A network of microphones can capture these events due to their distributed nature, picking up varying intensities of sound waves from different locations. Since the GPS coordinates of the nodes are known and the RMS values of the audio event are passed from the ESP32 nodes in the graph, we can calculate approximately the location via triangulation or the direction of arrival of the audio event.

Each node produces a graph of events, and the combination of different sensors’ reports are complementary with respect to a targeted event and can be merged into a single but more complete piece of information. Riots, massive attacks of low-flying drones, breach of border fences, conflicts involving multiple subjects can be considered a single incident with a distributed form, as their acoustic imprint typically unfolds over a broad area. In such dispersed scenarios, each node in the microphone network generates a graph representing detected audio events over time. By analyzing and parsing the paths within these multiple graphs, we can identify correlations and patterns that indicate the occurrence and progression of an event that has a distributive nature. The overlapping and sequential nature of audio signals across nodes helps to trace the propagation path of audio classes, revealing key details such as the geographic spread, intensity distribution, and temporal dynamics of the incident. This multi-graph analysis serves as a robust method for detecting and understanding incidents in dispersed forms, which are rarely addressed in the related research literature. The layers of abstraction can be extended further by clustering related incidents, enabling even higher-order interpretations.

## 3. Results

We conducted the following experiments: (A) we validated the system’s real-time response using data streams from both static and moving urban environments at the audio event level; (B) we evaluated the generated event graph for false alarms at the incident level; (C) we assessed the event graph for detection accuracy and missed identification of simulated hazardous incidents at the incident level; (D) we formally evaluated the system’s accuracy on a commercial movie by analyzing its audio stream for car crash detection, and (E) we measured execution speed across various platforms, including both edge and non-edge devices.

A. Accuracy at the audio event level

In this section we assess the accuracy of the system at the level of isolated events using audio snippets from real audio streams that do not belong to the AudioSet database.

### 3.1. Stationary Platforms

To assess the applicability of the discussed method in real-life scenarios, we installed three nodes submitting recordings to the base station through Wi-Fi. The nodes were located near busy streets to collect urban sounds. We had two assessment stages, one for the stationary version and one for moving platforms. First, we listened to 200 clips classified by the device and measured the classification accuracy of the system in a real-time flow of urban sounds. The human operator listened to the clips and decided if the label produced by the AST following the ontology of the AudioSet was correct. The correctness was assessed according to the highest-probability ranking (top one), or if the correct label was in the first two (top two) or top three classifier’s decisions. During this assessment, we were interested in all labels of the AudioSet ontology. The formal assessment of the AST has been reported in [27] and is also summarized in Section 2.2.2. What we are assessing in this work is the accuracy of the AST in real data that were impossible to use for its actual training.

### 3.2. Moving Platforms

The second test involved 200 recordings taken from moving platforms. We envision that in self-aware cities, microphones are installed on slowly moving platforms such as buses, trams, etc., and sample the audio scene of the city. The moving platforms do not have the dense time-resolution of a permanent station, but they sample the soundscape at large spatial scales, and the GPS coordinates of the moving platform can be inserted in the header of the filename, thus producing an audio track of urban soundscenes. We installed the nodes on cars moving around the city and stored the audio on the SD card of the nodes. Any recording deemed private was deleted prior to any processing but the vast majority of sounds were typical urban sounds that one encounters on a street walk (i.e., car sounds, voices, horns, sirens, bubble noise, animal sounds, etc.). In Table 2, we gather the results of automatically classified audio originating from microphones on stationary and moving platforms. Top one accuracy refers to the correctness of the prediction based solely on the class with the highest probability. In contrast, top two and top three accuracy compare the manual label to a set of including the second- and third-highest-ranked predictions as well, respectively. Moving platforms present a small degradation in performance due to the brevity of events and the Doppler shift that distorts the frequency content of audio classes recorded from microphones installed in moving platforms. However, in general, the AST is very accurate in recognizing the correct audio class in the wild.

B. Accuracy at the incident level

The incident category requires the integration of several, possibly different, audio events to be defined, e.g., car crash, burglary, domestic violence, etc.

### 3.3. Incidents of Special Interest

There are many potential applications for an audio surveillance system. To name a few, such a system could be used to detect patterns of public disturbances, monitor wildlife activity in urban areas, detect early signs of industrial hazards, for emergency response to natural disasters, and for crowd management in large gatherings. Depending on the application, there is a different audio context of what constitutes typical and atypical sound events. As a second evaluation stage, we have inserted in the real stream of audio feeds six simulated scenarios of atypical situations. The audio data simulating these rare situations have been realized using professional audio clips from the movie industry. Then, the whole database exported in JSON format was turned into a graph, and we made Python code that parses the graph and responds to various questions to gather evidence.

1. List all audio events 10 min prior to and after a gunshot. Gunshots and explosions are very brief events.
2. List all devices that picked up an explosion at 7.45 am and figured out the direction of arrival/location of the incident. Determine the blast’s direction of arrival and possibly the epicenter through analysis of the RMS values of each audio event.
3. Examine all nodes within a rolling time frame and present correlation of labels belonging to the incidence class that indicate the occurrence of an event having a distributive nature (e.g., drone attacks, riot).

Besides gathering evidence and examining situations of broad interest using LLM-fabricated code, we developed original Python code for three special cases of interest in which we want the system to work automatically. The approach is presented in Section 2, and the scenarios are as follows:

*Car accident:* The ‘primary_sounds’ class is composed of: ‘car’, ‘car passing by’, ‘motorcycle’, ‘truck’, ‘train’, ‘vehicle’, ‘bus’, ‘race car, auto racing’, ‘accelerating, revving, vroom’, ‘whoosh, swoosh, swish’. The ‘secondary_sounds’ class is: ‘breaking’, ‘shatter’, ‘smash’, ‘crash’, ‘bang’, ‘thump’, ‘thud’, ‘slam’, ‘glass’.

*Burglary:* The ‘primary_sounds’ class is composed of: ‘breaking’, ‘explosion’, ‘shatter’, ‘smash’, ‘crash’, ‘skidding’, ‘tire squeal’, ‘squeal’, ‘bang’, ‘thump’, ‘thud’, ‘glass’. The 

‘secondary_sounds’ class for this scenario is: ‘walk, footsteps’, ‘creak’, ‘buzzer’, ‘siren’, ‘alarm’, ‘whispering’, ‘slam’.

*Border control:* The ‘secondary_sounds’ class is for this application is: ‘gunshot, gunfire’, ‘explosion’, ‘burst, pop’, ‘machine gun’, ‘eruption’. The ‘secondary_sounds’ class is composed of: ‘screaming’, ‘groan’, ‘gasp’, ‘crying, sobbing’, ‘baby cry, infant cry’, ‘whimper’, ‘yell’, ‘slam’, ‘glass’.

*Domestic violence*: The only labels of this application are: ‘whimper’, ‘wail, moan’, ‘bellow’, ‘crying, sobbing’, ‘screaming’, ‘groan’.

Structured recordings of these incidents have been uploaded to the system, disrupting the normal flow of audio clips received by the baseline system. This evaluation is therefore semi-real, as the nodes operate within a real urban soundscape, but the flow of events has been intentionally interrupted by inserting recordings that simulate the three scenarios described earlier. These recordings are sourced from audio libraries commonly used in the movie industry.

We first assessed the JSON file of the annotation produced by AST for 15 days of real stream of urban audio scenes, for false alarms using the approach described in Section 2. We tested the detection of incidents of car crash, burglary, and breach of borders on a corpus of about 5000 events (i.e., audio clips) and none were referred to as such. Note that the keywords in each incident often appear in the JSON file (e.g., the label “Vehicle” about 500 times). We then generated 10 distinct realizations for each scenario and inserted this group of events in the real stream of data. Notably, all 30 events were successfully detected by the system, demonstrating the robustness of seeking coexisting labels from the AudioSet ontology and integrating evidence through a moving time window. Table 3 provides two examples of the system’s output. However, it should be noted that although the false alarms can be assessed by a real flow of data from nodes, detection accuracy and misses of these incidents cannot currently be evaluated against true cases due to their rare and hazardous nature.

### 3.4. Assessment at the Incident Level

The movie *Crash* (1996) by David Kroneberg has a duration of 1:40:02. Our primary objective was to detect car crashes in this movie from the audio alone. We extracted the audio part and we downsampled it to a 16 kHz sampling rate and mp3 mono format. Then, we treated the whole movie as if it were a real stream of audio data and we manually timestamped the ground truth of car crashes using the picture content (see Table 4). This procedure allowed us to derive precise error metrics for hazardous events that would not have been possible to derive from real-time streams. Note that a movie includes complex audio scenes, music and dialogue that can complicate the audio scape. We manually identified 16 scenes, of which 8 were visually verified as car crashes—incidents where both the visual evidence and the audible impact confirmed that a crash occurred—and 8 scenes depicted dangerous driving practices without any impact. In Table 1, the “Positive” column records the timestamps of the car crash scenes, while the “Negative” column contains events that, despite featuring vehicle sounds, skidding, accelerating noises, and tires squealing, do not culminate in an impact. A crash was only documented when there was both visual confirmation of the incident and audible verification of an impact, so that the negative dataset comprised a particularly challenging set of cases that closely mimicked accidents without actually resulting in one. We classified the negative cases in Table 1 as ‘Careless driving’, whereas the positive column was labeled ‘Car crash incidents’. As shown in Table 4, this dataset contains 17 scenes/events—8 positive cases and 9 negative (the time gaps between the positive cases).

#### 3.4.1. Our Approach

**Figure 6:**
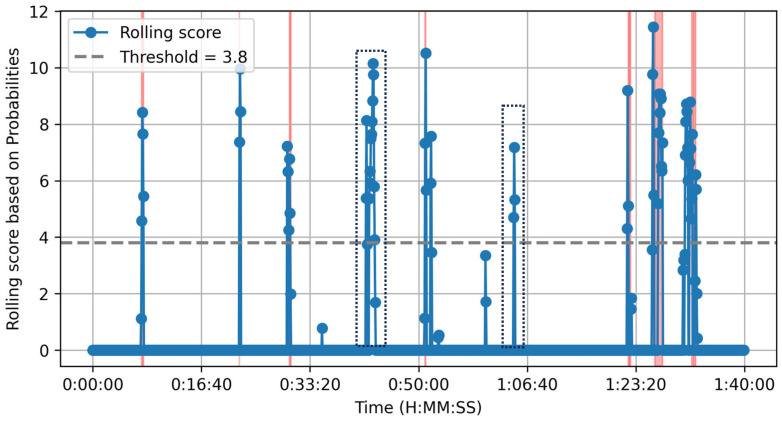
Our approach. The pink/red areas indicate scenes where a car crash occurs. The blue vertical lines represent the rolling incident score. An incident is detected when the contextual moving window includes a vehicle-related sound and an impact, potentially followed by an audible aftermath (e.g., glass breaking), though the latter is not mandatory. The dotted squares highlight false alarms.

Our Python algorithm processes a rolling window of two 10 s audio chunks with a 50% overlap, using the top five AST labels for classification. The algorithm correctly identifies all eight true positives (TPs) and produces two false positives (FPs) (see Figure 6 and compare the peaks to the timestamps in Table 4). As shown in Figure 6, our approach delivers clear-cut decisions for car crash incidents. Despite making decisions on a per 10 s basis, it generated only two false alarms throughout the entire movie. The two misclassified scenes contained vehicle-related audio and skidding sounds but did not result in an actual crash.

#### 3.4.2. An LLM Approach

**Figure 7:**
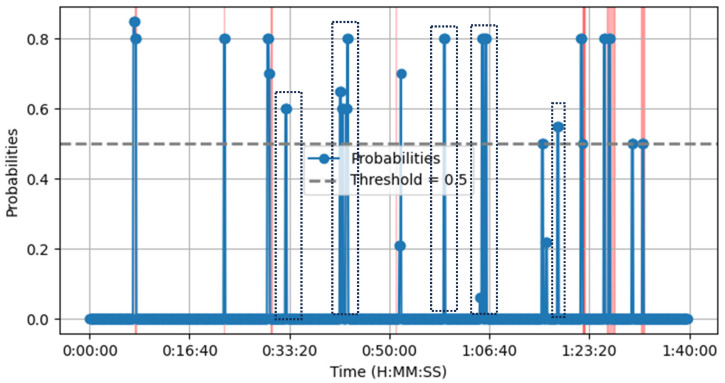
Llama-3.1-8B q8 receives the audio transcription of the *Crash* movie using the AST and decides if a car crash takes place every 10 s. The pink/red areas indicate scenes where a car crash occurs. The blue vertical lines represent the rolling incident score. The dotted squares highlight false alarms.

The LLM approach utilizes the same AST labels as our proposed method to ensure a fair comparison. As shown in Figure 7, we processed the same data using LLaMA-3.1-8B with DSPy prompting. While the LLM produced results closely aligned with our approach, it generated a higher number of false alarms. Specifically, it correctly detected all eight crash scenes but also produced five false positives. Notably, due to the model’s size and the need for per-chunk decision-making across an entire movie, we did not use the largest LLaMA model. We quantized the model to reduce its memory footprint and computational requirements, making it more efficient for deployment on resource-constrained hardware. We used the q8 version (quantization to 8-bit integers INT8 format) instead of using the more accurate standard 16-bit (FP16) or 32-bit (FP32) floating-point precision. The temperature was set to a low point of 0.2 to ensure that we obtained more deterministic, repetitive, and conservative responses. The top_p was set to 0.95, meaning that the model made selections from the top 95% of the probability mass.

#### 3.4.3. Metrics

We assessed the accuracy of the system using several metrics: accuracy, precision, recall, and F1-score. These metrics allowed us to validate the accuracy of our approaches. All scores exceeding the threshold were deemed positive, and if they were inside the manually tagged time span of a positive scene, then they were deemed true positive or otherwise false alarms.

Accuracy is the ratio of correctly predicted instances to the total number of instances, providing an overall measure of classification performance.

$$Accuracy=\frac{TP+TN}{TP+FP+TN+FN}$$

Precision quantifies the proportion of true-positive predictions out of all positive predictions made by the model, indicating the exactness of positive identifications.

Recall, also known as sensitivity, measures the ratio of true-positive predictions to all actual positives, reflecting the model’s ability to capture all relevant cases.

$$Recall=\frac{TP}{TP+FN}$$

The F1-score, calculated as the harmonic mean of precision and recall, offers a balanced metric that accounts for both false positives and false negatives.

$$F1=2\times \frac{\mathrm{Precision}\times \mathrm{Recall}}{\mathrm{Precision}+\mathrm{Recall}}$$

These metrics, gathered in Table 5, are essential for a comprehensive evaluation of classifier performance, especially in scenarios with class imbalances where reliance on accuracy alone may lead to misleading conclusions.

### 3.5. Deployment on Edge Devices

The recognition network has been fully deployed on off-the-shelf MCUs. The primary constraints for MCU deployment are execution speed and memory requirements for storing the transformer’s weights. In this work, we did not focus on optimizing power consumption. We tested the system on a Raspberry Pi 4, Raspberry Pi 5, and an i5 laptop without a GPU. Table 6 summarizes the processing and upload times for classifying a 5 s audio file.

### 3.6. Error Analysis and Mitigation Approaches

We have examined an extensive number of collected recordings for which the flagged incidents are false alarms or misses, and hereinafter, we discuss the main sources of errors we discovered and why they occurred. Regarding false alarms, we wish to express the following:

1. Depending on the context, the class targeted by an AST may not always correspond to an actual incident. A real example observed during experimentation occurred during the transition from 2024 to 2025, when the sensors detected the sound of fireworks, which lasted for about 10 min. Although some classifications were correctly made for “Fireworks” or “Firecracker” sounds, as these classes are included in the AudioSet, many others were identified as “Artillery fire” and “Gunshot, gunfire”. In adverse weather, we observed that the “thunder” class may be confused with “eruption” or “explosion”. The same was observed when distant airplanes were passing over. The audio modality can be fooled, as cries of joy/surprise can be misinterpreted as cries of pain and breaking of things does not necessarily imply an unlawful action. As explained in Section 2, we rely on a hierarchy of sounds within a time frame to classify an incident, and this approach considerably reduces the false alarm cases.
2. In moving platforms, Doppler shift distorts the spectral content of audio that does not match the spectral distribution of audio that the transformer was trained, on as the majority of sounds in the AudioSet dataset were taken from stationary microphones.
3. Sounds at a distance lose their high-frequency content and may be erroneously classified as other types of impulsive sounds, e.g., “Door”.
4. The recordings often contain overlapping classes of sounds because the urban sound scene can become complex. Therefore, an irrelevant sound, e.g., a bird’s vocalization, can receive a higher probability than a target signal. The fact we retain the first three possible classifications mitigates the impact of this.
5. Sometimes critical incidents evolve quickly (e.g., a car crash can take less than five seconds, and a gunfire incident may take even less time). This is why we interpret a collection/cluster of targeted labels within a timeframe as an incident and not just a static, single label.

By tailoring the AudioSet ontology to the specific application context, we can constrain the number of possible sound classes, enhancing precision. For instance, when the aim is to detect car crashes, all labels related to musical instruments or genres in the AudioSet ontology can be excluded. Our frontend allows the user to select the audio classes the application anticipates. The algorithm ranks the probabilities of all classes and skips to the next permissible class if a detected class is deemed irrelevant. In urban environments or border control scenarios, many sound types are unexpected and can be excluded by the user as irrelevant.

The core idea of this work is to treat audio as text and integrate labels into a rolling working memory to determine the presence of hazardous incidents, and we do not implement optimization for peak performance. Several steps could be taken to enhance the system’s overall efficiency, including fine-tuning the transformer model with application-specific data, applying augmentation techniques during training, and utilizing audio source separation and enhancement methods.

## 4. Discussion

In our vision of self-aware environments in smart cities, a large network of discreet microphones, cameras, and various other sensors (e.g., proximity and passive infrared sensors) are integrated seamlessly in its infrastructure and continuously monitor and register human activity. These sensors can work collaboratively to create a dynamic, real-time map of events within the city. When an accident or an unlawful action occurs, the system uses algorithms to parse and filter an immense amount of automatically annotated data that reduces the original dimensionality of the audio–visual data by orders of magnitude. Each sensing node contributes an abstraction of local events in the form of a graph that connects people, objects, and actions over time and space. The integration of all individual graphs constitutes the state graph. The latter graph enables authorities or automated systems to analyze incidents, trace the path to its origins, and resolve it either in time or retrospective. The state graph can be traced throughout the time but also within all nodes under a fixed timeframe, thus answering different questions related to localized and distributed incidents. Such environments not only enhance public safety but also provide a framework for accountability and rapid incident resolution in urban settings. In this work, the audio modality was investigated within the realm of transformers and edge devices.

### 4.1. Ethical Issues

Cameras and microphones are types of sensors that are often perceived as invasive, particularly when monitoring involves human subjects. Consequently, privacy concerns must be carefully addressed to meet strict requirements, including transparency, robustness, and accountability. It is essential to consider the potential ethical, safety, and regulatory aspects of the work, including its objectives, methodology, and the possible implications of its outcomes. Our arguments are based on the following premises:

- Threatening situations: Crimes and terrorist acts in large urban areas are not hypothetical scenarios but real threats that actually occur and demand special attention and preventive measures. The knowledge that public spaces are secured through intelligent monitoring systems is expected to deter such acts.
- Legal and common practices: Surveillance is generally lawful and widely accepted in places like stores, banks, agencies, and airports, where the need for heightened security justifies the installation of video cameras.
- Unattended surveillance: Autonomous surveillance systems are significantly less invasive than traditional monitoring by individuals. These systems eliminate human interference in interpreting sensor data and prevent data broadcasting at any stage of the inference process. This reduces the risk of unauthorized circulation of personal information, thereby aligning with growing public demands for privacy protection.
- Primary function: The main role of unattended surveillance is to detect critical situations in real time and deliver timely warning messages to authorized personnel. It does not engage in any uncontrolled or autonomous actions beyond this scope. The application scenarios emphasize security services in residential areas, workplace environments, and borders, prioritizing citizen safety.
- Political context: The decision to implement acoustic surveillance in public spaces, along with the laws governing this service, is inherently political and shaped by the cultural values of a regime. However, this paper does not address political considerations; it focuses exclusively on applied research.

### 4.2. Improvements

We tried to expand the probability estimation by finding semantic distances between user queries and AST classification results using WordNet version 3.1 via NLTK 3.8.1 and spaCy 3.8.5 but the vectors did not yield useful semantic distances for these domain-specific classes. We attribute this fact to the general utility of semantic classifiers that are not strictly defined in the context of audio surveillance, and this is why we built a hand-crafted hierarchy of AudioSet labels on a per-case scenario. This reduces reliance on external semantic tools and ensures relevance.

Instead of rule-based systems, we could consider using a machine learning approach, but this would require simulations, as these audio classes are rare in reality. If one had labeled incidents (examples of true accidents and false alarms), one could train a model to predict whether a cluster of sounds represents a true incident. This would shift the burden of manually grouping events with semantic similarity as queries and it would learn patterns from the domain data.

The system is designed to analyze events by constructing a graph representation of their relationships, enabling a range of advanced analyses. As discussed in [33], various approaches connect audio with language modeling, with a central idea of treating audio as if it were language. These models achieve this by fusing text and audio clips during training, allowing the system to generate captions for the input audio stream when prompted under operational conditions.

While these approaches can be integrated into our platform, audio captioning can produce a multitude of correct outputs [33] and this may not be compatible with the sensitive nature of our application. It is essential first to observe how this highly active area of research evolves to a steady state and aligns with the applied nature of our work. Additionally, a careful assessment of the error patterns in these models, particularly in relation to the critical concept of security, is necessary.

In our point of view, the system we suggest is more straight forward. The graph of events produced allows for visualization of event connections, identification of clusters of related events, and detection of patterns, such as chains of events, that might indicate significant occurrences. The approach can be adapted to different temporal dynamics by using a dynamic windowing mechanism. This window can expand when specific patterns emerge, or the analysis can be split into multiple overlapping time windows to accommodate event sequences of varying durations. For instance, some accidents unfold over extended periods, while others occur in seconds, necessitating this flexible temporal analysis.

The platform could also incorporate advanced tools for filtering and drilling down into specific events or categories, supported by a centralized event storage solution. A time-series database, such as ElasticSearch or InfluxDB, can be employed to store events efficiently. These databases are optimized for indexing data, enabling rapid querying and effective visualization of event relationships.

To further enhance analysis, the system could employ shared AI models capable of handling diverse inputs (usually audio–visual). A modular classification model is trained to function across different domains, ensuring flexibility. Additionally, graph neural networks are utilized to establish correlations between events, such as linking acoustic anomalies to potential security breaches. This combination of modular AI models, dynamic graph analysis, centralized storage, and human feedback creates a robust framework for detecting, analyzing, and acting on critical events across domains.

## 5. Conclusions

Despite valid privacy concerns, many surveillance systems have been deployed to monitor and ensure public safety. These systems generate vast amounts of data that require automated filtering and analysis, either for real-time detection of dangerous situations or for offline information retrieval. Our current objective is to identify audio and respond to unusual but critical actions, such as violent acts, accidents, or potential threats, in real time.

We combine AI with edge devices and audio to filter the immense flow of recorded events per day, per node, and abstract hazardous situations from clusters of audio events. The primary goal is to provide a decision-support interface that enhances the performance of human experts by integrating machine-generated predictions with expert insights. The focus is on leveraging surveillance solutions as active, real-time tools rather than merely retrospective forensic instruments.

Audio surveillance has currently reached a mature stage and holds significant potential to transform urban safety by creating interconnected, security-conscious environments in today’s densely populated and complex cities. This is achieved by collecting precise and timely field information, including audio events, their class distribution, and their criticality. In this work, the central idea is to treat audio as text and create a rolling window of labels for audio events. Once composite keywords are detected within the rolling window, an accumulation of scores begins. Alternatively, when an LLM is used, the labels serve as anchor points, and the LLM fills in the details to infer a decision based on a prompt. An incident is detected if the system integrates enough evidence from audio events that either persist over time or are highly characteristic (e.g., gunshots).

## Figures and Tables

**Table 1:** JSON format of annotated soundscapes. We depict two events with their associated timestamps and the first three highly ranked probabilities.

“event_id”: “evt_593”, “timestamp”: “2024-11-24 12:01:57”, “audio_events”: [ (“class”: “Screaming”, “probability”: 0.55), (“class”: “explosion”, “probability”: 0.1), (“class”: “Gasp”, “probability”: 0.09)], “related_to”: “evt_585”), ( “event_id”: “evt_595”, “timestamp”: “2024-11-24 12:01:58”, “audio_events”: [ (“class”: “Gunshot, gunfire”, “probability”: 0.22), (“class”: “Burst, pop”, “probability”: 0.19), (“class”: “Explosion”, “probability”: 0.14)], “related_to”: “evt_593” )

**Table 2:** Classification accuracy (%) of a stream of audio events in urban environments. “Stationary” refers to microphones near traffic and “moving” platform refers to recording devices attached externally to cars. The human observer compares if the true label is in the top 1–3 labels provided by the AST classifier.

Platform	Top 1	Top 2	Top 3
Stationary	88.8	97.2	99.3
Moving	84.3	95.6	97.1

**Table 3:** Automatically produced incidents and the evidence upon which they have been based. The indicators are the labels produced by the AST.

Type: Gunfire Detected Time Window: 2024-11-24 12:01:57 to 2024-11-24 12:01:58 Related Events: [‘evt_595’, ‘evt_593’] Indicators Used: [‘screaming’, ‘gunshot, gunfire’, ‘explosion’, ‘burst, pop’] Scores: (‘total_primary’: ‘0.65’, ‘total_secondary’: ‘0.55’) Type: High-level Event Detected Time Window: 2024-11-24 12:06:22 to 2024-11-24 12:06:27 Related Events: [‘evt_613’, ‘evt_619’, ‘evt_614’, ‘evt_621’, ‘evt_605’] Indicators Used: [‘screaming’, ‘gunshot, gunfire’, ‘burst, pop’, ‘groan’] Scores: (‘total_primary’: ‘0.41’, ‘total_secondary’: ‘1.70’)

**Table 4:** Timestamps in H:M:S format of positive crashes and car-related audio events involving skidding, accelerating, tires squealing and horns that do not end with a car crash (negative column). Some events contain several consequent impacts.

Event	Positive	Negative
1	0:07:30–0:07:46	0:18:45
2	0:22:28–0:22:30	0:35:10
3	0:30:10–0:30:24	0:42:40–0:43:15
4	0:51:00–0:51:06	0:51:51–0:51:57
5	1:22:09–1:22:19	0:58
6	1:22:25–1:22:30	1:00:15–1:00:24
7	1:26:15–1:27:27	1:20:55
8	1:31:57–1:32:00	1:25:55–1:26:00

**Table 5:** Accuracy metrics using our algorithm and Llama-3.1-8B (LLM).

	Accuracy		Precision		Recall		F1-Score	
Incident	Ours	LLM	Ours	LLM	Ours	LLM	Ours	LLM
Crash	0.882	0.706	0.8	0.615	1.0	1.0	0.889	0.762

**Table 6:** Processing time of a 5 sec recording on different devices.

	Raspberry Pi 4	Raspberry Pi 5	i5 Laptop
Time (s)	14	4.6	1.2

## Data Availability

The dataset of audio events is available from the corresponding author upon reasonable request.

## Abbreviations

The following abbreviations are used in this manuscript:

RMS	Root, mean square value
CA	Certificate Authorities
CCTV	Closed-Circuit Television
SSL	Secure Sockets Layer
HTTPS	Hypertext Transfer Protocol Secure
AST	Audio Spectrogram Transformer
IoT	Internet of things
MQTT	Message Queuing Telemetry Transport
NVS	Non-volatile storage
TLS	Transport Layer Security
GPRS	Message Queuing Telemetry Transport
PSRAM	Pseudostatic read-only memory
STFT	Message Queuing Telemetry Transport
VIT	Vision Transformer
SQL	Structured Query Language
JSON	JavaScript Object Notation
AAC	Advanced Audio Coding
MP3	Moving Picture Experts Group Audio Layer III
DoS	Denial of service
ACL	Access Control List

## Appendix A. Prompt Syntax Using DSPy

System message [32]:

Your input fields are:

1. ‘question’ (str): Question to be answered

2. ‘context’ (str): additional information to make decisions

3. ‘chunks’ (str): List of chunks format chunk_id

Time range of the recording

sounds in chunk: Probability of sound

End of chunk

Example: Chunk1

0:00:00–0:00:10

car: 0.99

crash: 0.01

end of chunk

Chunk 2

0:00:10–0:00:20

car: 0.99

crash: 0.01

end of chunk

Your output fields are:

1. ‘step_of_thought’ (str): Describe the steps of the decision-making process, indicating the chunk id and time intervals of the sounds considered to contribute to the decision, the reason they contribute to the decision, and the sequence of sounds based on the content instructions that contribute to the decision.

2. ‘summary’ (str): Explain your decision about incident in a single sentence

3. ‘answe’ (str): is there an incident described in the question. Only Yes or No

4. ‘probability’ (float): Probability of incident 0–1

Respond with the corresponding output fields, starting with the field ‘[[ ## step_of_thought ## ]]’, then ‘[[ ## summary ## ]]’, then ‘[[ ## answer ## ]]’, then ‘[[ ## posibility ## ]]’ (must be formatted as a valid Python float), and then ending with the marker for ‘[[ ## completed ## ]]’.

In adhering to this structure, your objective is as follows: You are an audio analyst. You must predict whether the event described in the question is caused by the sounds in the following audio recordings. You are given context with additional information to help you decide whether an event will occur. You are given a list of recordings at specific time intervals. Each recording contains the track id | track recording time range and a list of the most likely sounds detected in that track, next to the sound is the probability with which the sound was detected. Consider whether the event described in the question is caused by the sounds of the recording.

User message:

[[ ## question ## ]]

Is there an incident of a car accident or car crash?

[[ ## context ## ]]

To detect a car accident or traffic accident or car crash, the following conditions must be met: Car Sounds: Detectable sounds must include car-related noises such as engine sounds, mechanical sounds, skidding, tire squealing, or similar. The probability of these car sounds must exceed 0.1 to be considered valid. Collision Sounds: There must be clear evidence of a collision, such as a crash, bang, explosion, breaking glass, shattering, or similar sounds. The probability of these collision sounds must exceed 0.1 to be considered valid.

Temporal Sequence: A car sound with a probability exceeding 0.1 must occur before a collision sound with a probability exceeding 0.1. Without a collision sound, a car accident cannot be confirmed. Note: Skidding or tire squealing alone is insufficient to confirm a collision, as the accident might have been avoided. Contextual Consistency: The car sound must precede the collision sound, either in the same audio chunk or in a previous chunk.
